# Detection of hepatitis C virus RNA in saliva of patients with active infection not associated with periodontal or liver disease severity

**DOI:** 10.1186/1471-2334-14-72

**Published:** 2014-02-10

**Authors:** Francisca Sosa-Jurado, Verónica L Hernández-Galindo, Daniel Meléndez-Mena, Miguel A Mendoza-Torres, Fernando J Martínez-Arroniz, Verónica Vallejo-Ruiz, Julio Reyes-Leyva, Gerardo Santos-López

**Affiliations:** 1 Laboratorio de Biología Molecular y Virología, Centro de Investigación Biomédica de Oriente, Instituto Mexicano del Seguro Social, Km 4.5 Carretera Federal Atlixco-Metepec, Metepec, Puebla CP 74360, México; 2 Maestría en Ciencias en Investigación Clínica, Escuela Superior de Medicina y Homeopatía, Instituto Politécnico Nacional, Distrito Federal, México; 3 Servicio de Gastroenterología, Hospital de Especialidades, Unidad Médica de Alta Especialidad, Centro Médico Nacional General de División Manuel Ávila Camacho, Instituto Mexicano del Seguro Social, Puebla, Puebla, México; 4 Facultad de Estomatología, Benemérita Universidad Autónoma de Puebla, Puebla, Puebla, México

**Keywords:** Periodontal disease, Viral load, Gingivitis prevalence, Risk factors, Liver disease severity

## Abstract

**Background:**

Hepatitis C virus (HCV) is mainly transmitted by parenteral route, being blood transfusion and intravenous drug use the most frequent risk factors. However, it has been suggested that there are other routes of transmission. There are several studies where HCV RNA has been detected in saliva of patients infected with HCV, and epidemiological studies have proposed the dental treatments as possible risk factors for HCV transmission. The purpose of this study was to detect the presence of HCV RNA in saliva of patients with active infection and associating with periodontal or liver disease.

**Methods:**

Patients with quantifiable HCV-RNA in serum were enrolled in the study. Periodontal disease was assessed using the modified gingival index (MGI). Presence of dental plaque was assessed with the use of disclosing tablets. Patients were clinically and laboratory evaluated to identify the stage of liver disease, the HCV RNA was determinate in saliva by nested RT-PCR. To determine associations between different parameters univariate and multivariate analysis were used.

**Results:**

A total of 45 patients were included. Of these patients, 21 (46.6%) had hepatitis, 23 (51.1%) had cirrhosis and one patient (2.4%) presented hepatocellular carcinoma (HCC). Viral loads in serum ranged from 2.31–6.68 log IU/ml with a mean of 5.46 log IU/ml (95% CI 5.23–5.70). HCV RNA was positive in saliva of 29 patients (64.4%) and was not detected in 16 (35.6%). For univariate analysis three independent variables were associated with the detection of HCV-RNA in saliva: gender, viral load and dental plaque and multivariate analysis only one independent variable viral load >5.17 log IU/mL remained significantly associated with the detection of HCV in saliva (*p* = 0.0002). A statistical difference was observed when viral load was analyzed, log 5.85 IU/mL (95% CI 5.67–6.02) for patients with HCV in saliva vs. log 4.77 IU/mL (95% CI 4.35–5.19) for patients without HCV in saliva (*p* = 0.0001). The detection of HCV-RNA in saliva was more frequent in patients with relatively high serum viral loads.

**Conclusion:**

HCV-RNA in saliva was associated with the level of serum viral load but not with periodontal or liver disease severity.

## Background

Hepatitis C virus (HCV) infection is an important public health concern with an estimated 160 million persons (prevalence ~2.35%) infected worldwide [1]. HCV is the causative agent of chronic hepatitis, cirrhosis, and hepatocellular carcinoma (HCC). Thus, HCV causes 27% and 25% of all cirrhosis and HCC cases, respectively [2].

HCV is mainly transmitted by parenteral route, and the more frequent risk factors are blood transfusion before the availability of tests to donors screening and intravenous drug use. HCV transmission is also associated with other risk factors including unprotected sexual practices, multiple sex partners, tattooing, piercing and history of either surgery or intra-familial hepatitis [3].

Frequent diagnosis of HCV infection in patients without any parenteral risk factor suggests the existence of other transmission routes [4]. At respect, HCV RNA has been detected in the saliva of HCV-infected patients, [5-8] and epidemiological studies proposed dental treatment is another probable risk factor.

A contributing factor for transmission of HCV in saliva is the presence of periodontal disease [9]. Accordingly, severe gingivitis is characterized by a constant bleeding accompanied by an increased secretion of gingival crevicular fluid (GCF), which may be potential sources of HCV [5] and the salivary glands [10-12]

Gingivitis prevalence reaches 84% in adult males [13] and 54% in females [14] in some regions of Mexico. Therefore, in the present work we determined the presence of viral RNA in saliva samples of a group of patients with active hepatitis C in order to identify whether detection of salivary HCV is associated with the presence of periodontal disease, severity of liver disease, or the amount of viral RNA in peripheral blood.

## Methods

### Patients

Forty-five patients who received medical care at the Gastroenterology Service of the High Specialty Medical Unit of Mexican Institute for Social Security in Puebla, Mexico were enrolled in the study. Inclusion criteria were patients of both genders, age range 18–70 years, abnormal alanine aminotransferase (ALT) levels, positive to anti-HCV antibodies and detectable HCV RNA in serum (viral load >1.77 log IU/ml). Patients were clinically and laboratory evaluated to identify stage of liver disease. As part of the diagnostic procedures, prothrombin time and platelet counts were performed. An epidemiological questionnaire was applied to evaluate risk factors, and written informed consent was obtained from each participant.

Patients co-infected with human immunodeficiency virus (HIV) and/or hepatitis B virus (HBV), patients who had undergone antiviral treatment, edentate patients and those with oral ulcers were excluded from the study.

The study was performed following ethical regulations approved by Local Research and Health Ethics Committee #2101 of Mexican Institute Social Security (Protocol: R-2008-2101-10) and in accordance with the Declaration of Helsinki.

The sample size (*n*) was calculated according to Hernandez Samperi et al., 2010 [15], using the formula: *n = (n’/(1 + n’/N))*, where *n’* is calculated as *s*^2^/*V*^2^, *s*^2^ = *p* (1- *p*) and *V*^2^ is the square of standard error, while *N* is the number of patients without treatment; *p* is the prevalence (%) calculated in previous studies reporting the positivity to HCV in saliva [5,7]. The substitution in this formula was carried out with the following values: *N* = 80, *se* = 0.05, and *p* = 0.3.

### Measurement of modified gingival index

Periodontal status was evaluated using the Modified Gingival Index (MGI). Results were reported as healthy (0 to < 0.1), mild (0.1 to < 1.0), moderate (1.0 – 2.0) and severe (> 2.0) [16,17].

### Determination of dental plaque

In order to assess the amount of dental plaque, disclosing tablets were used to stain the teeth. Tooth pigmentation was then compared with a dental chart and results were reported as good < 20%, Acceptable 20% – 40%, and poor > 40% [18].

### Sample collection and RNA extraction in saliva

Saliva was collected in sterile glass jars. Samples were aliquoted and transferred to 1.5 ml sterile tubes and stored at -80ºC until use. Total RNA was extracted from 200 μl of saliva using Trizol reagent (Invitrogen, Carlsbad, CA, USA) following conventional procedures. RNA concentration and quality were determined by spectrophotometry and visualized by 1% agarose gel electrophoresis with ethidium bromide.

### HCV RNA detection in saliva

HCV-RNA was amplified using a nested reverse transcription-PCR with the two sets of primers corresponding to the 5′UTR, as reported previously [19,20]. Reverse transcription and the first round of PCR (25-μl reactions) were performed using One-Step RT-PCR with Platinum Taq (Invitrogen). Program parameters were 55°C for 30 min, 94°C for 2 min, and 40 cycles of 94°C for 30 sec, 56°C for 30 sec, and 72°C for 30 sec. The second round of PCR (25 μl reactions) was performed using BioMix (Bioline, London, UK) and 2 μl of first-round PCR product. Thermal cycler conditions were 94°C for 2 min and 40 cycles of 94°C for 30 sec, 60°C for 30 sec, and 72°C for 30 sec. The 305 and 251 bp PCR products were analyzed with 1% agarose gel electrophoresis. This technique has a detection limit of 50 IU/ml, comparable with commercial tests used for qualitative detection of HCV genome [21,22]. A fragment of the housekeeping gene cyclophilin was amplified for each sample as internal control for RNA extraction and RT/PCR reaction, using primers and conditions previously published [23]. In all test series, known HCV negative and positive serum controls were included and the samples were managed with the same precautions during taking, transport and processing.

### Determination of serum viral load

Total RNA in serum samples was extracted by an automated process performed in BioRobot 9604 (Qiagen, Hilden, Germany) and viral loads were determined by means of the COBAS Taqman HCV Test v.2.0 (Roche Molecular Systems, Indianapolis, IN).

### HCV detection and genotyping in serum

The HCV RNA in serum samples was extracted using QIAamp RNA Viral Mini Kit (Qiagen, Valencia, CA) and was amplified using a nested RT-PCR with the two sets of primers corresponding to the 5′ UTR, as reported previously [18,19]. HCV genotype testing was performed by line probe assay INNO-LiPA version 2.0 (Innogenetics, Ghent, Belgium).

### Data analysis

For continuous variables, averages, frequencies and percentages were calculated. Univariate analysis was performed using χ^2^ or Fisher's exact test to detect independent variables associated with detection of viral RNA in saliva. The variables with *p* values < 0.1 were introduced in multivariate analysis and, after that, those with *p* values <0.05 were considered as independently associated with viral RNA detection. Pearson’s correlation between MGI and percentage of plaque was also applied. And to analyze differences between viral loads was used Mann–Whitney U test. All statistical analysis was performed using Statgraphics Centurion XV v. 15.2.06 (Stat Point Technologies, Inc., Virginia, U.S.A.).

## Results

The minimal sample size determined was n = 39 patients. In this study we studied a total of 45 patients, of which, 19 (42.2%) were male and 26 (57.8%) were female. Mean age was 45.7 years (95% CI: 42.4–49.0). Twenty one (46.6%) patients had chronic hepatitis without clinical signs of cirrhosis, 23 (51.1%) had various grades of cirrhosis and one (2.4%) patient presented HCC. Viral load in serum was in the range of 2.31–6.68 log IU/ml with a mean of 5.46 log IU/ml (95% CI 5.23–5.70). Prothrombin time was in the range of 45–104% with a mean of 79% (95% CI 73–84). Platelet count was in the range of 5 × 10^4^–3.0 × 10^5^ per mm^3^ with a mean of 1.55 × 10^5^ per mm^3^ (95% CI 1.30 x 10^5^–1.80 x 10^5^) (Table 1). HCV genotype was determined in 45 patients included in the study, 17 patients had HCV 1a (37.7%), 20 had 1b (44.4%), 1 had 2a (2.2%), 3 had 2b (6.8%) and 4 had 3a (8.9%) (Table 1).

**Table 1:** Epidemiological characteristics of HCV infected patients

**General characteristics**	***n *** **(%)**
Male/female	19 (42.2 )/26 (57.8)
Mean age	45.7 years (95% CI 42.4–49.0 years)
Risk	
Blood transfusion	25 (55.5)
Multiple sex partners	6 (13.3)
History of surgery	2 (4.4)
Dental care	1 (2.2)
Intravenous drug user	2 (4.4)
Unidentified source of infection	9 (20)
Liver disease status	
Active hepatitis	21 (46.6)
Cirrhosis	23 (51.1)
Hepatocellular carcinoma	1 (2.3)
Laboratory data	
Range serum viral load (log IU/ml)	2.31–6.68
Mean serum viral load (log IU/ml)	5.46 (95% CI 5.23–5.70)
Range prothrombin time (%)	45–104
Mean prothrombin time (%)	79 (95% CI 73–84)
Range platelet count (μl)	5.3 × 10^4^–3.0 × 10^5^
Mean platelet count (μl)	1.55 × 10^5^ (1.30 × 10^5^–1.80 × 10^5^)
HCV genotype and subtype	
1a	17 (37.8)
1b	20 (44.4)
2a	1 (2.2)
2b	3 (6.7)
3a	4 (8.9)

**Figure 1:**
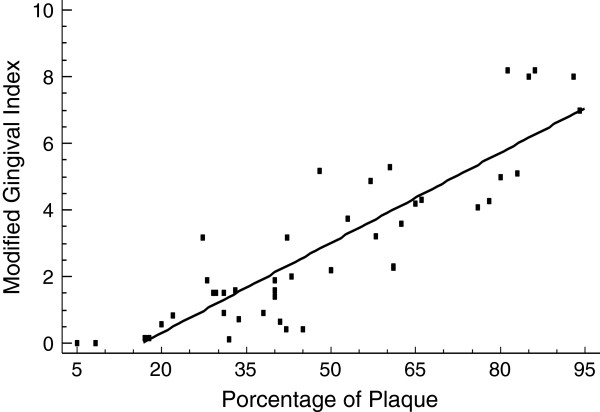
**The Pearson test was used to show the correlation between variables.** There was a significant positive correlation between the percentage of plaque and the modified gingival index (r =0.88; p = 0.0094).

Four patients presented a good level of dental plaque (8.9%), but this was just acceptable in 16 (35.6%) and unacceptable in 25 (55.6%) patients (Table 2). The degree of the MGI found in patients with active HCV infection was as follows: healthy (0.0–0.1) in three (6.7%) patients, mild (0.2–1.0) in 10 (22.2%) patients, moderate (1.1–2.0) in seven (15.5%) patients, and severe (>2.0) in 25 (55.6%) patients (Table 2). A positive correlation (r = 0.88, p = 0.0094) between MGI and dental plaque was found (Figure 1).

**Table 2:** Dental study data and HVC detected in saliva

**MGI**	***n *** **(%)**
Healthy (0.0–0.1)	3 (6.7)
Mild (0.2–1.0)	10 (22.2)
Moderate (1.1–2.0)	7 (15.5)
Severe (>2.0)	25 (55.6)
Dental plaque (%)	
Good (≤20%)	4 (8.9)
Acceptable (20%–40%)	16 (35.6)
Unacceptable (>40%)	25 (55.6)
Salivary HCV	
Detected	29 (64.4)
Not detected	16 (35.6)

MGI, modified gingival index.

HCVRNA was detected in the saliva of 29 (64.4%) patients and was not detected in 16 (35.6%) patients (Table 2). According to univariate analysis, three independent variables were associated with the detection of HCV RNA in saliva: gender, viral load and acceptable level of dental plaque. Indeed, 16/19 men (84.2%) vs. 13/26 women (50%) were positive to HCV in saliva (*p* =0.001); 27/31 (87%) patients with a viral load >5.17 log IU/Ml were positive to HCV in saliva vs. 2/14 (14.3%) patients with viral load <5.17 log IU/mL (*p* = 0.0000); and 15/20 (75%) patients with an acceptable level of dental plaque were positive to HCV in saliva vs. 14/25 (56%) patients with an unacceptable level of dental plaque (*p* = 0.07) (Table 3). Univariate analysis also showed that age, liver disease severity, MGI, prothrombin activity, platelet counts, and viral genotype were not associated with detection of HCV RNA in saliva. At respect, salivary HVC was detected in 15/22 (49%) patients ≤ 45 years old vs. 14/23 (51%) patients >46 years old (*p* = 0.17); in 13/22 (59%) patients with cirrhosis vs. 16/23 (69.5%) patients without cirrhosis (*p* = 0.358). There were 9/13 (69.2%) patients with platelet counts <10^5^/mm^3^ vs. 20/32 (62.5%) patients with platelet counts >10^5^/mm^3^ (*p* = 0.652). There were 8/12 (66%) patients with activity prothrombin <70% vs. 21/33 (63%) patients with activity prothrombin >70% (*p* = 0.703). There were 9/13 (69%) patients with moderate to severe gingivitis (MGI >1.0) vs. 20/32 patients with healthy gums (MGI <1.0) (*p* = 0.66). With respect to HCV genotype 24/37(64.7%) patients had genotype 1 vs. 5/8(62%) with other genotype (p =0.484) (Table 3).

**Table 3:** Factors associated with salivary Hepatitis C Virus detection

	**Univariate analysis**		**Multivariate analysis**	
**Factor**	**OR (95% CI)**	***p***	**OR (95% CI)**	***p***
Gender (male)	9.91 (7.74–12.6)	0.001	_____________	_____
Age (>46 years)	0.53 (0.37–0.54)	0.173	_____________	_____
Cirrhosis	0.56 (0.46–0.67)	0.358	_____________	_____
MGI (>1.0)	1.35 (1.09–1.67)	0.666	_____________	_____
Dental plaque (<40%)	3.21 (2.51–3.99)	0.070	_____________	_____
Serum viral load (>5.17 log IU/ml)	40.5 (30.8–53.0)	0.0000	25 (18.8–32.5)	0.0002
Prothrombin time (<70%)	1.22 (0.98–1.44)	0.703	_____________	_____
Platelet count (<10^5^ mm^3^)	0.74 (0.61–0.89)	0.652	_____________	_____
Viral genotype	0.54 ( 0.29-0.68)	0.481	_____________	_____

MGI, modified gingival index.

**Figure 2:**
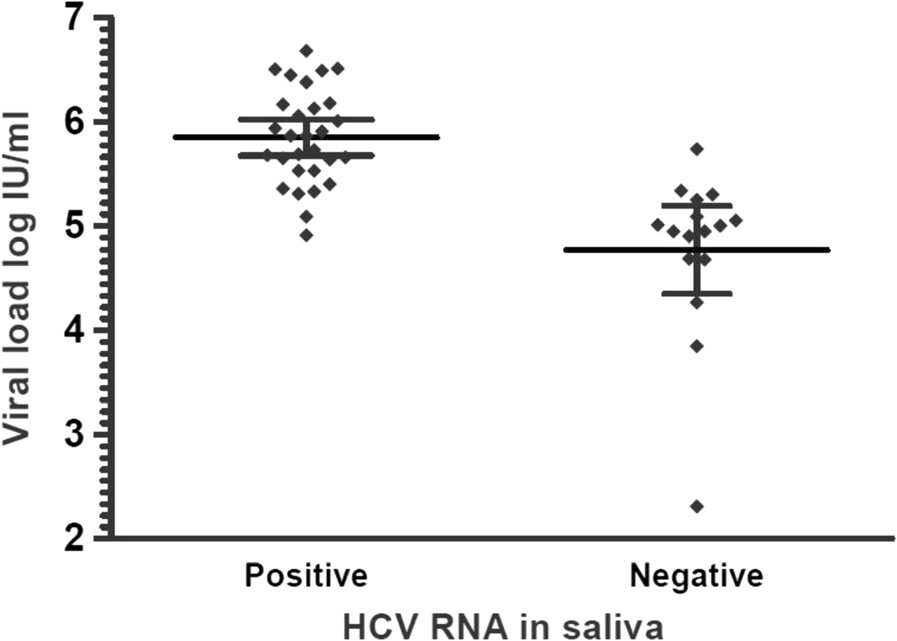
**The Mann–Whitney test was used for serum viral loads comparison of patients with or without salivary HCV RNA (p = 0.0001).** The graph shows the mean and the 95% confidence interval.

The variables with a *p* value <0.1 were introduced in a multivariate analysis and only one independent variable, viral load >5.17 log IU/mL, remained significantly associated with HCV detection in saliva (*p* = 0.0002) (Table 3). To corroborate that viral load was associated with the presence of HCV in saliva; patients were grouped in the basis of their positive or negative result of salivary HCV detection. A statistical difference was observed between viral load means, log 5.85 IU/mL (95% CI 5.67–6.02) for patients with HCV in saliva vs. 4.77 IU/mL (95% CI 4.35–5.19) for patients without HCV in saliva (*p* = 0.0001) (Figure 2).

## Discussion

In this study we determined the presence of HCV in saliva of patients with active hepatitis C in order to identify some associated factors. The role of oral cavity fluids in the transmission of HCV is controversial. Several studies reported the presence of HCV RNA in saliva, but the infectivity of salivary HCV particles has not been confirmed. Then, there is no solid data supporting that the presence of HCV in saliva is an effective HCV transmission route.

In addition, epidemiological studies show the limited transmission of salivary HCV [4]. Potential sources of HCV within saliva includes GCF, leukocytes that migrate through the epithelium to the gingival groove [5], salivary glands [10,11] and peripheral blood mononuclear cells from bleeding tissues [12]. Therefore, we hypothesized that the presence of periodontal and gingival disease may be a contributing factor for virus dissemination. However, in our study and that of others, salivary HCV has been detected both in patients with healthy and diseased periodontium [5,6,8]. In this regard we did not find any association between the degree of gingival inflammation and viral RNA detection in saliva. There was neither significant difference of salivary HCV between patients with healthy gums (9/13, 69%) and those with gingival disease (20/32, 62.5%). It was observed a positive correlation between MGI and percent plaque (Figure 1), coinciding with other studies demonstrated that the severity of periodontal disease varies depending on the quantity and quality of the presence of biofilm [24]. We found that the main factor associated with the presence of HCV in saliva was the high viral load in serum (>5.17 log IU/ml) as has been found in some other studies [5,7]. In our study, 27/29 (93.1%) patients with viral RNA in saliva had high viral loads; however, 4/16 (25%) patients with a negative result in saliva were also within the range of high viral load (5.17–5.60 log IU/ml).

In our work we determined two other clinical parameters in patients, in order to obtain other data related with the severity of liver disease. Prothrombine time and platelets counts are important parameters may be associated with bleeding of bucal cavity, therefore may be associated with a higher probability of infection through saliva, however, none of two parameters was statistically associated with presence of HCV RNA in saliva.

The prevalence of salivary HCV differed with the source of the samples, study population, and method of viral RNA extraction and detection. In our study the prevalence of HCV in saliva was 64.4% in patients with active infection. Some studies reported prevalence values of 31–100% in saliva samples [5,7] and 38–85% in oral and gingival groove fluid samples [5,8]. Another study reported 52% of salivary HCV in a group of patients coinfected with HIV [25].

It is notable that, in our study, 20% of patients had an unidentifiable source of infection as has been found in other studies [26,27]. Several epidemiological studies of HCV performed in Mexican people that included blood donors, [28-30] open population, [31,32] and patients with cirrhosis [33] mentioned dental treatment as a probable risk factor. Other studies found that living with a family member infected with HCV [34,35] or suffering from liver disease [33,36,37] was an associated risk factor. Although it is known that multiple factors contribute to HCV transmissibility for saliva [5,38] and viral loads are very low in this [39]. The presence of HCV in saliva is to draw attention mainly in patients with active infection and high viral loads, patients who require guidance on their oral health by dentist. On the other hand there have been very favorable changes in the practice of infection control procedures among dentists. Although, not all are well familiar with infection control procedures [40,41]. These results should be interpreted with caution. Since the domestic nonparenteral transmission of HCV occurs [42,43], but transmission mechanisms have not been established.

## Conclusion

Detection of HCV in saliva was associated with a relatively high viral load in serum but not with the level of periodontal disease or liver disease severity.

## Abbreviations

HCV: Hepatitis C virus; RNA: Ribonucleic acid; MGI: Modified gingival index; GCF: Gingival crevicular fluid; ALT: Alanine aminotransferase; HIV: Human immunodeficiency virus; HBV: Hepatitis B virus.

## Competing interests

The authors declare that they have no competing interests.

## Authors’ contributions

FSJ, VVR, JRL and GSL participated in the study design, molecular tests, in the data analysis and in drafting and discussing the manuscript. VLHG and FJMA performed the evaluation of periodontal disease using the modified gingival index and presence of dental plaque and sampled the patients. DMM and MAMT received the patients in the Gastroenterology Service and assessment clinical and laboratory studies and participated in the analysis of clinical epidemiological data. All authors read and approved the final manuscript.

## Pre-publication history

The pre-publication history for this paper can be accessed here:

http://www.biomedcentral.com/1471-2334/14/72/prepub
